# Surfactant Treatment Shows Higher Correlation Between Ventilator and EIT Tidal Volumes in an RDS Animal Model

**DOI:** 10.3389/fphys.2022.814320

**Published:** 2022-04-20

**Authors:** Yoon Zi Kim, Hee Yoon Choi, Yong Sung Choi, Chae Young Kim, Young Joo Lee, Sung Hoon Chung

**Affiliations:** ^1^ Department of Pediatrics, College of Medicine Kyung Hee University, Seoul, South Korea; ^2^ Department of Obstetrics and Gynecology, College of Medicine Kyung Hee University, Seoul, South Korea

**Keywords:** electronic impedance tomography, respiratory distress syndrome, premature infant, neonatal intensive care unit (NICU), mechanical ventilator, alveoli collapse, homogeneity

## Abstract

Neonatal respiratory distress syndrome (RDS) is a condition of pulmonary surfactant insufficiency in the premature newborn. As such, artificial pulmonary surfactant administration is a key treatment. Despite continued improvement in the clinical outcomes of RDS, there are currently no established bedside tools to monitor whether pulmonary surfactant is effectively instilled throughout the lungs. Electrical impedance tomography (EIT) is an emerging technique in which physiological functions are monitored on the basis of temporal changes in conductivity of different tissues in the body. In this preliminary study, we aimed to assess how EIT tidal volumes correlate with ventilator tidal volumes in an RDS animal model, namely untreated, surfactant-treated, and normal control rabbit pups. Tidal volumes were measured simultaneously on an EIT system and a mechanical ventilator and compared at different peak inspiratory pressures. The linear correlation between tidal volumes measured by EIT and by ventilator had an *R* ^2^ of 0.71, 0.76 and 0.86 in the untreated, surfactant-treated, and normal control groups, respectively. Bland–Altman analysis showed a good correlation between the measurements obtained with these two modalities. The intraclass correlation coefficients (ICC) between ventilator tidal volume and EIT tidal volume were 0.83, 0.87, and 0.93 (all *p* < 0.001) in the untreated, surfactant-treated, and normal control groups, respectively, indicating that the higher ICC value, the better inflated status of the lung. In conclusion, we demonstrated that EIT tidal volume correlated with ventilator tidal volume. ICC was higher in the surfactant treated group.

## Introduction

Neonatal respiratory distress syndrome (RDS) is a condition of pulmonary surfactant insufficiency in the premature newborn; without treatment, morbidity and mortality increase during the first 2 days of life. Administration of artificial pulmonary surfactant reduces surface tension of the alveoli and improves functional residual capacity by expanding the collapsed alveoli (Knudsen and Ochs, 2018).

Despite continued improvement of the clinical outcomes in RDS, there is a scarcity of bedside tools that can be used to monitor the state of the instilled surfactant throughout the lungs, excepting chest x-ray and blood gas analysis. Electrical impedance tomography (EIT) is an emerging technique in which ventilation is monitored on the basis of temporal changes in conductivity of different tissues in the body. With small currents, conducting electrodes attached to the skin can analyze body composition during ventilation without radiation exposure (Metherall et al., 1996; Adler and Boyle, 2017; Kallio et al., 2019). However, its application to neonatal RDS is not yet established.

We analyzed the calculated tidal volumes using the EIT technique. The aim of this study was to determine how pulmonary surfactant administration affects tidal volume parameters measured by mechanical ventilator and EIT in a preterm rabbit model of RDS.

## Materials and Methods

### Animals

According to the ARRIVE guidelines, we obtained approval from the Institutional Animal Review Board of Kyung Hee University Hospital (KHMC-IACUC-2017-026). Zoletil® (15 mg/kg) was used to induce sedative anesthesia of the mother rabbit (intravascular injection, marginal ear vein) and each pup (intramuscular injection, unilateral thigh muscle) before procedures. We harvested preterm rabbit pups on day 27 (D27) of gestation and term pups on day 31 (D31) *via* caesarean section of pregnant New Zealand white rabbits. The study groups were as follows: 1) Untreated (preterm pups), 2) surfactant-treated (preterm pups), and 3) normal controls (term pups). Soon after delivery, we performed a tracheostomy procedure on the pups using a 24G intravenous catheter and applied a small animal ventilator (VentElite, Harvard Apparatus, Holliston, MA, United States) set to the pressure-controlled mandatory ventilation mode (Supplementary Figure S1). The treated group were given 100 mg/kg of Curosuf ® (Chiesi Farmaceutici, Parma, Italy) by the intratracheal route, immediately after application of the ventilator.

### Electrical Impedance Measurement

We used the KHU Mark2.5 EIT system, a non-commercial prototype designed by the Impedance Imaging Research Center of Kyung Hee University. Since the rabbit pups were small and had an average weight of 43.06 ± 14.75 g and a chest circumference of 7.64 ± 0.61 mm, we prepared a specialized bed that had an electrode interface (Supplementary Figure S1). The pups were placed in the interface with sixteen spring-loaded pin electrodes that surrounded the chest, just below the level of the forelimbs. The zigzag electrode attachment and a measurement protocol robust against noise and electrode attachment position error were selected because of the small chest circumference of the preterm pups (Graham and Adler, 2007). To collect time series of EIT images at 50 frames/s, 16 electrode leads were connected in two layers at the perimeter of the thorax of each rabbit pup.

**FIGURE 1:**
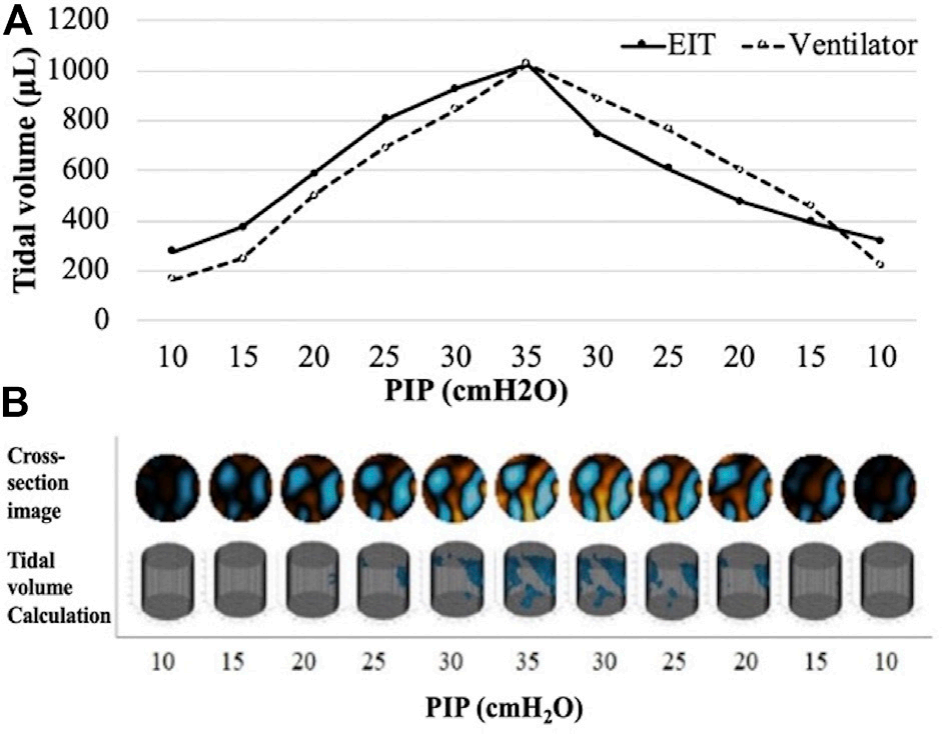
An example of tidal volume measurement and EIT images in a subject from preterm treated group **(A)** Pressure-volume curves of ventilator tidal volume and EIT tidal volume. **(B)** The reconstructed chest cross-section EIT according to PIP change. PIP, peak inspiratory pressure.

The EIT measurement was undertaken during the following ventilator maneuver: An initial respiratory rate set at 120 breaths/min and positive end-expiratory pressure of 5 cm H_2_O (Basoalto, et al., 2021; Ferrini, et al., 2021; Joelsson et al., 2021). Over 120 s, PIP was increased from 10 to 35 cm H_2_O in 5 cm H_2_O increments (stepwise inflation) and thereafter returned in 5 cm H_2_O steps back to the baseline (stepwise deflation). EIT images were reconstructed from the difference in voltage data measured between adjacent electrode pairs using the 3D GREIT algorithm (Figures 1A,B) (Adler and Lionheart, 2006). At each PIP level, the tidal volumes (V_T_) measured by the ventilator were recorded, and the EIT signals were transformed into aeration area and calculated tidal volumes. The calculation was done by subtracting the individual pixel values of relative impedance changes in EIT during each PIP step. Finally, the local impedance change was plotted in the selected regions of interest.

### Statistical Analysis

The volume of air in the lungs and the tidal volume, was the major determinant of thoracic impedance change. Within each group, we calculated the differences in tidal volume (Diff V_T_) measured by the ventilator and EIT during PIP changes as follows:

$$\text{Diff }V_{T}\;\left(\text{µL}\right)=\text{Vent }V_{T}\;-\text{EIT }V_{T}$$

The relationship between ventilator tidal volume (Vent V_T_) and EIT tidal volume (EIT V_T_) was assessed by Spearman’s correlation and linear regression. Agreement between Diff V_T_ in the same PIP was analyzed by Wilcoxon signed rank test with paired test and equivalence test. In addition, the agreement was confirmed through the Bland–Altman plot. The intraclass correlation coefficient (ICC) was calculated for each group, and the ICCs were compared between different groups using Kruskal-Wallis test. Mann-Whitney U test was used in between-group comparisons. Statistical significance was set at *p* < 0.05 and SAS version 9.4 (SAS Institute Inc., Cary, NC, United States) as well as the R4.0.4 program was used for analyses.

## Results

Twenty three newborn pups were harvested from 6 mother rabbits. Five pups were excluded as a result of early death (*n* = 2) or measurement failure due to pneumothorax, which was recognizable by sight (*n* = 3). Among 18 pups, 3 were term, 9 were untreated preterm and 6 were surfactant treated preterm pups. We obtained 198 tidal volume sets in total (99 in treated, 66 in untreated and 33 in term group). One tidal volume set was made from 120 s of the specific PIP level.

### Assessment of Changes in Tidal Volume and Electrical Impedance Tomography

**FIGURE 2:**
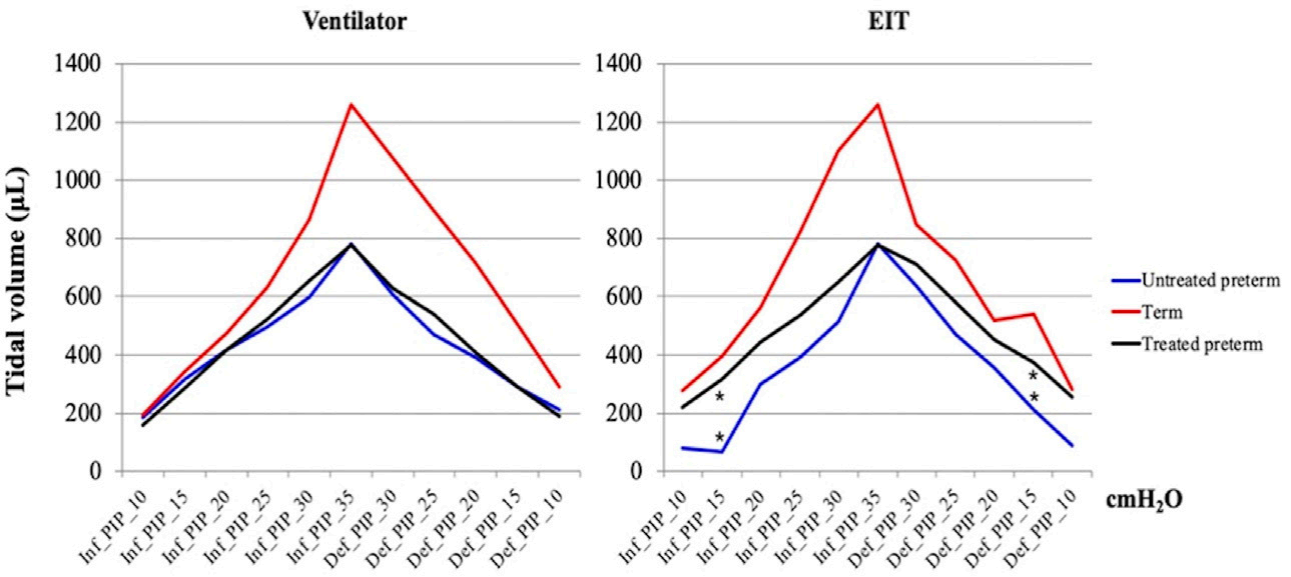
Pressure-volume curves. The graphs were based on the median values of the untreated preterm group (*n* = 9), surfactant treated group (*n* = 6), and term group (*n* = 3). Maximum and minimum values are shown in Table 1. * means statistical significance between the untreated preterm group and surfactant treated preterm group (*p* = 0.018, 0.026, Mann-Whitney U test). PIP, peak inspiratory pressure; Inf, inflation; Def, deflation.

We obtained pressure-volume curves according to inflation and deflation pressures. Table 1 and Figure 2 show the median values in the curves of Vent V_T_ and EIT V_T_. There was a significant difference between the untreated and surfactant-treated preterm pups in both inflation and deflation curves at PIP of 15 cm H_2_O (*p =* 0.018, 0.026).

**TABLE 1:** Measured tidal volumes according to stepwise inflation and deflation.

Ventilator^a^		Untreated preterm (*n* = 9)		Treated preterm (*n* = 6)		Term (*n* = 3)	
		Median	Range	Median	Range	Median	Range
Inflation peak inspiratory pressure (cmH_2_O)	10	186	(32–709)	161	107–397	194	110–266
	15	316	(110–997)	288.5	(232–553)	342	(188–396)
	20	419	(160–1,252)	419	(338–681)	477	(266–552)
	25	496	(214–1,467)	523	(444–788)	631	(370–812)
	30	599	(370–1754)	655	(545–913)	865	(600–1,097)
	35	781	(604–1929)	776	(705–1,026)	1,261	(916–1,435)
Deflation peak inspiratory pressure (cmH_2_O)	30	607	(499–1726)	628	(547–890)	1,076	(788–1,253)
	25	471	(318–1,519)	539	(444–766)	896	(682–1,071)
	20	393	(214–1,334)	408	(340–626)	717	(552–864)
	15	289	(134–1,157)	292.5	(237–496)	506	(396–604)
	10	210	(31–944)	189	(154–368)	291	(214–344)

**EIT** ^**b**^		**Untreated preterm**		**Treated preterm**		**Term**	
		**Median**	**Range**	**Median**	**Range**	**Median**	**Range**
Inflation peak inspiratory pressure (cmH_2_O)	10	81.8	(10.5–735.7)	222.2	(154.4–293.0)	277.2	(208.3–316.2)
	15	65.2^c^	(17.9–541.0)	316.2^c^	(257.3–386.8)	397.3	(247.6–562.0)
	20	299.8	(29.8–833.1)	443.4	(317.9–587.5)	564.0	(308.7–825.7)
	25	391.9	(51.1–1,114.0)	536.45	(470.4–804.4)	819.9	(375.5–1,028.0)
	30	515.9	(190.3–1,632.5)	650.12	(591.2–925.9)	1,102.9	(654.9–1,109.9)
	35	781.0	(632.0–1,632.5)	776.0	(705.0–1,026.0)	1,261.0	(825.6–1,444.0)
Deflation peak inspiratory pressure (cmH_2_O)	30	637.1	(542.0–1,594.7)	709.8	(540.4–914.1)	848.8	(721.0–1,257.9)
	25	468.9	(325.5–1929.0)	579.9	(439.3–729.9)	723.3	(625.8–1,079.6)
	20	354.9	(56.4–1926.1)	454.5	(319.6–517.9)	519.6	(485.2–870.3)
	15	212.3^c^	(17.9–1754.6)	375.0^c^	(226.2–454.5)	541.5	(371.8–618.3)
	10	88.89	(4.9–1812.7)	253.7	(149.6–332.1)	282.9	(223.1–350.8)

a Tidal volumes were recorded as shown by the small animal ventilator panel, according to peak inspiratory pressure changes.

b Tidal volumes were calculated through the EIT, signals analysis.

c Means statistical significance between untreated preterm group and surfactant treated preterm group (*p* = 0.018, 0.026, Mann-Whitney U test).

### Correlation Analysis of Tidal Volume Measured by the Ventilator and Electrical Impedance Tomography

**FIGURE 3:**
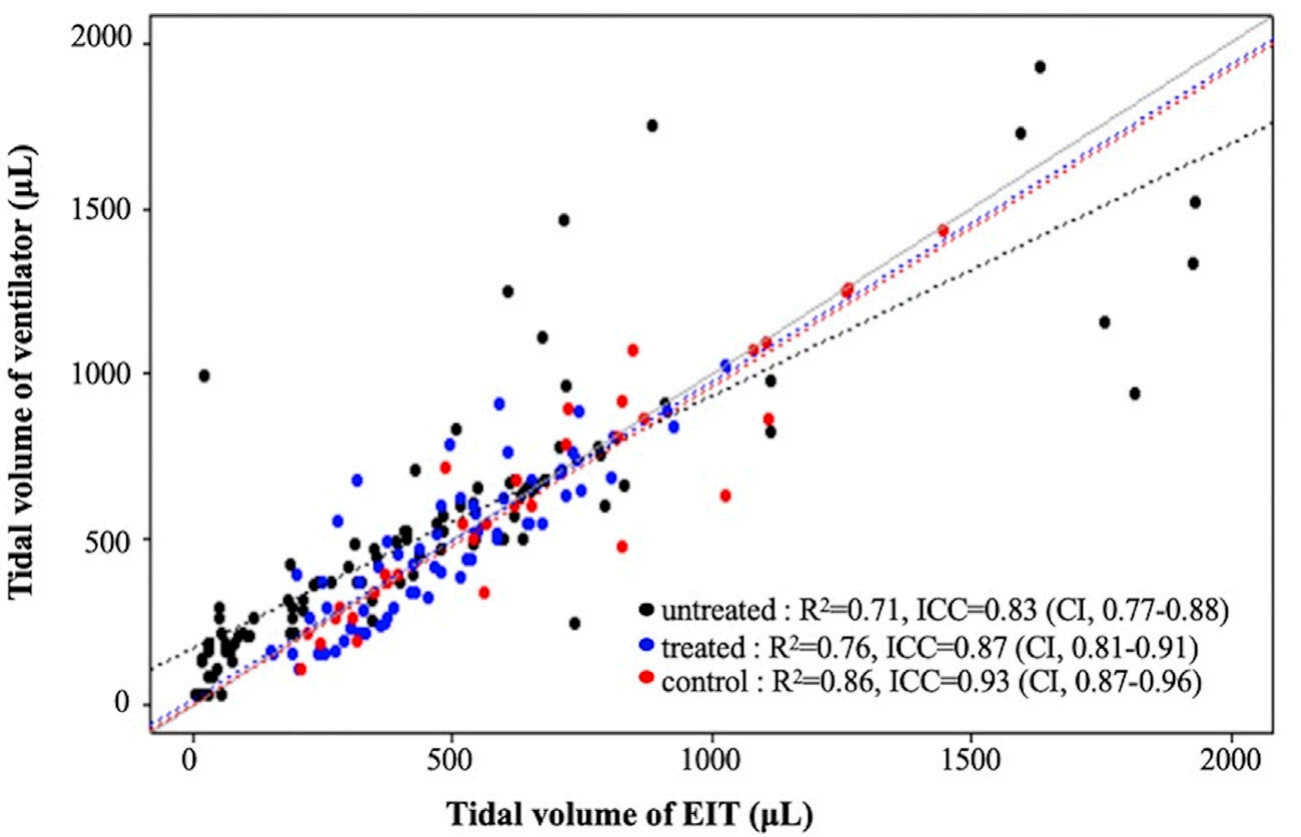
Linear regression analysis and intraclass correlation coefficient (ICC) of tidal volumes determined by the ventilator and EIT V_T_ in each group.

**FIGURE 4:**
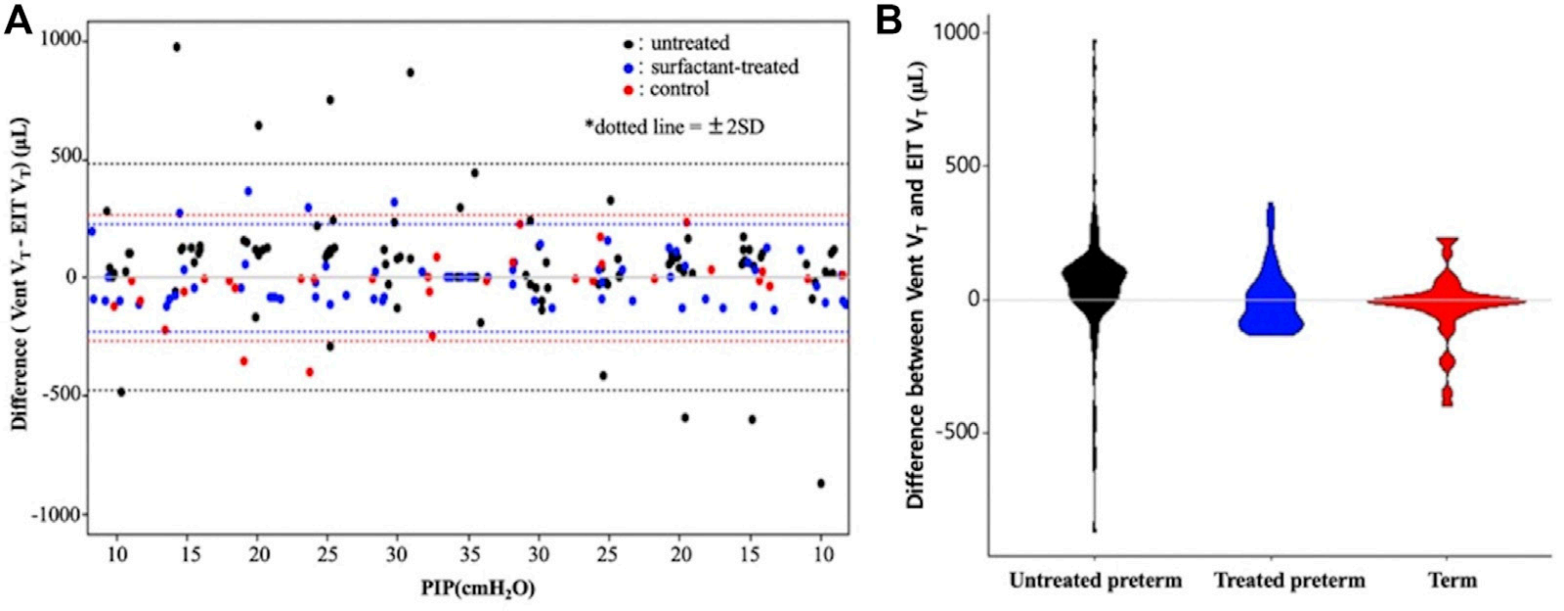
A Bland–Altman plot **(A)** and violin plot **(B)** showing differences between the ventilator and EIT with the 95% limits of agreement. PIP, peak inspiratory pressure; Vent V_T,_ ventilator tidal volume; EIT V_T_, EIT tidal volume.

An increase in PIP increased the tidal volume and lung impedance. A highly positive correlation between ventilator tidal volume and EIT tidal volume was found in all 18 pups; linear regression equations for each group are shown in Figure 3. The determination coefficients (*R* ^2^) of all groups were greater than 0.7, showing good correlation between ventilator and EIT. Moreover, the ICC between Vent V_T_ and EIT V_T_ was 0.85 (*p* < 0.001) in all groups, indicating very good agreement. The ICC values of each group were 0.83 (*p* < 0.001), 0.87 (*p* < 0.001), and 0.93 (*p* < 0.001) in the untreated, surfactant-treated, and normal control groups, respectively. ICCs of inflation PIP 10, 20, 25 cm H_2_O (*p* < 0.001) and deflation PIP 20, 15 and 10 cm H_2_O (*p* < 0.001) were significantly different between untreated and surfactant treated groups. The Diff V_T_ values at different PIP were analyzed by the Bland–Altman plot and the violin plot chart (Figure 4). The mean Diff V_T_ was 72.50 μL (range, 14.61 to 109.88 μL) in the untreated preterm group, −21.76 μL (−31.87 to 24.51 μL) in the surfactant-treated preterm group, and −7.88 μL (−71.46 to 21.96 μL) in the normal control group.

## Discussion

In our study, New Zealand white rabbit pups were used as a model of both premature lung physiology as well as RDS pathology. Rabbit pups harvested on D27 correspond to preterm births with RDS and those harvested on D31 correspond to normal term births (Kikkawa et al., 1968; Choi et al., 2017). Human infant RDS is a disease of prematurity. Its incidence is 92% at 24–25 weeks’ gestation, 88% at 26–27 weeks, 76% at 28–29 weeks, and 57% at 30–31 weeks of gestation (Sweet et al., 2013). As pulmonary surfactant is not ready to function in RDS infants, the mainstay of treatment is artificial surfactant instillation through the trachea. It recruits alveolar volume and hence increases lung residual functional capacity by reducing surface tension in the alveoli and equilibrating the uneven pressures of different parts of the lungs, present in RDS.

The purpose of this study was to determine whether EIT can generate some sort of practical benefit in the bedside treatment of artificial surfactant in the rabbit pup model of RDS. Overall, our findings show good correlations and agreement analysis between the measurements of tidal volumes that were obtained from the two modalities, small animal ventilator and EIT. The most interesting finding was of higher ICC values in the surfactant treated group (0.87) compared to the untreated group (0.83), not to mention the highest ICC values in the term group (0.93, Figure 3) and its narrow distribution in the treated group (Figure 4). Although our study design precludes before and after intervention comparisons, it is feasible to assume that surfactant instillation could increase the ICC from a lower pretreatment state, in mechanically ventilated pups. We attempted for pre- and post-intervention comparisons, however, the preterm pups could not survive long enough for the measurements. As such, we simplified our model into 3 groups instead of performing longitudinal analyses.

In terms of the ICC differences, a possible explanation could be that unlike untreated pups, unevenly inflated or collapsed alveoli were minimized in the surfactant treated preterm pup lungs, as opposed to the untreated pups, eventually empowering the agreement analysis. Accordingly, the higher ICC values and *R* ^2^ may indicate more recruited alveoli.

EIT technology is already an emerging option in various ICU environments such as adult type acute respiratory distress syndrome, pneumothorax, atelectasis, and pleural effusion (Bodenstein et al., 2009; Burkhardt et al.,. 2013; Davies et al., 2019; Jang et al., 2019; Tomicic and Cornejo, 2019). In neonatal settings, there are several publications regarding infants with RDS (Frerichs et al., 2001; Chatziioannidis et al., 2011; Miedema et al., 2011; Chatziioannidis et al., 2013). Unlike these studies, however, we analyzed tidal volumes from two different modalities and observed higher ICC in the surfactant treated group. We suggest that EIT technology may be a promising option as a real-time bedside monitoring tool in mechanically ventilated RDS infants, but further study is needed.

The most challenging problem of the rabbit pup model in our study, especially the preterm model, is that they are very small (42.1 ± 14 g in this study). They cannot be cannulated, blood gas analyses are not available, and imaging studies are limited. However, the premature lung physiology that encompasses RDS pathology is a great strength that allows for testing of artificial surfactant efficacy (Almlén et al., 2010; Otsubo and Takei, 2002).

This study has several limitations. First, our study lacks assessment of pre and post effects of surfactant administration. The pups were small and premature, and they could not survive long enough to endure another set of procedures. Second, this was a preliminary study using animal subjects, conducted with a small sample size. In addition, we regarded all measured tidal volumes to be a result of intact ventilation without any leakage. Finally, we compared only the tidal volume sets without the regional ventilation data of EIT.

## Conclusion

In conclusion, in this preliminary animal study, we observed good correlation of tidal volumes between Vent V_T_ and EIT V_T_. Furthermore, there were better ICC and Diff V_T_ in surfactant treated RDS group than in the untreated group. EIT can detect an improvement in lung ventilation in surfactant treated and term pups, compared to untreated rabbit pups.

## Data Availability

The original contributions presented in the study are included in the article/Supplementary Material, further inquiries can be directed to the corresponding author.

## Supplementary Material

Supplementary Figure S1 Experimental setting of EIT and a small animal ventilator with a rabbit pup.

Supplementary Figure S2 The specialized bed designed by the Kyung Hee University Impedance Imaging Research Center (IIRC).
