# Supplementary figures and images for: Surfactant Treatment Shows Higher Correlation Between Ventilator and EIT Tidal Volumes in an RDS Animal Model
Source: Front Physiol. 2022 Apr 20;13:814320. doi: 10.3389/fphys.2022.814320 (PMC9065679; doi:10.3389/fphys.2022.814320)

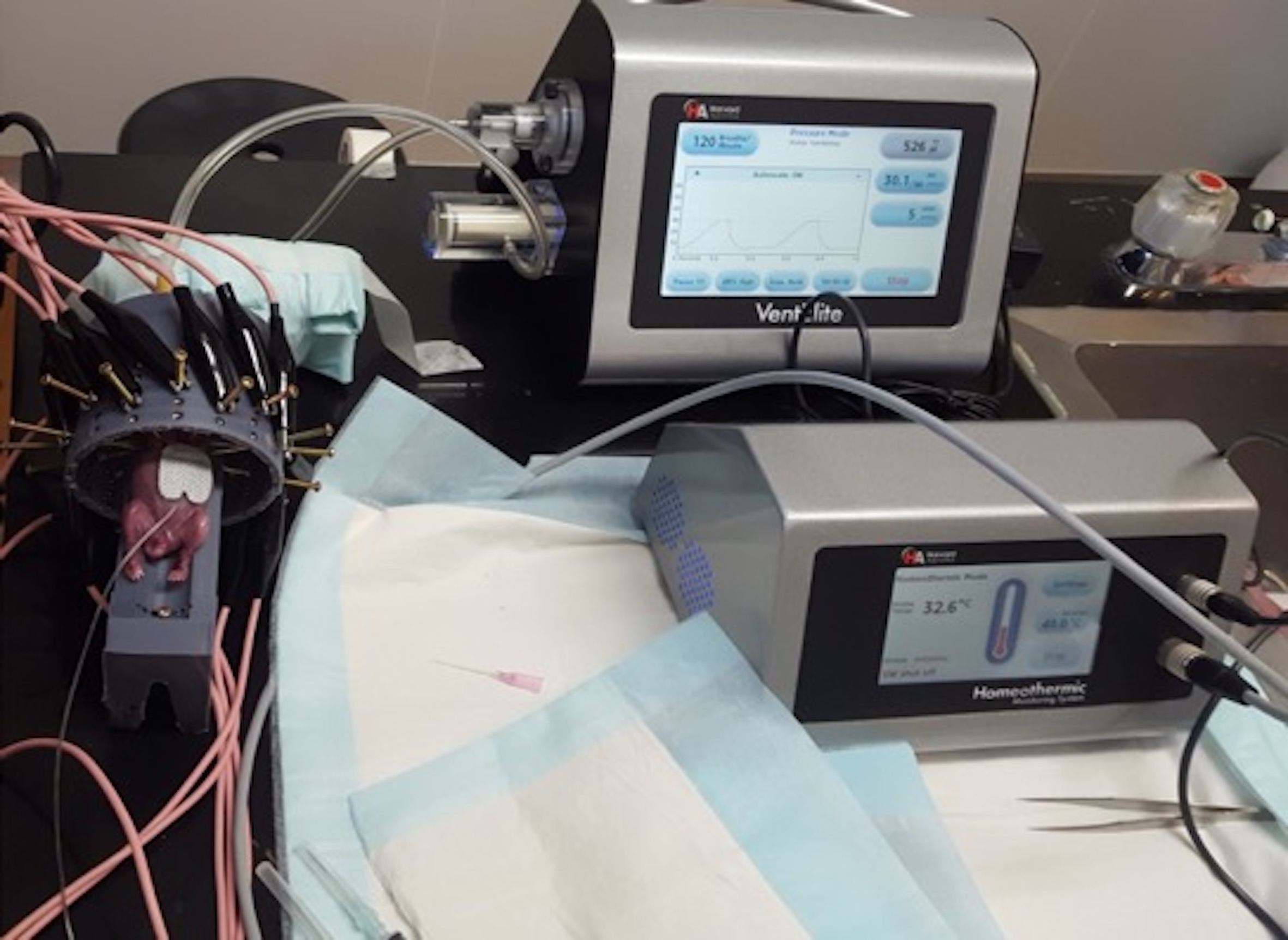

Supplement: Supplementary file 1 [file Image1.JPEG]

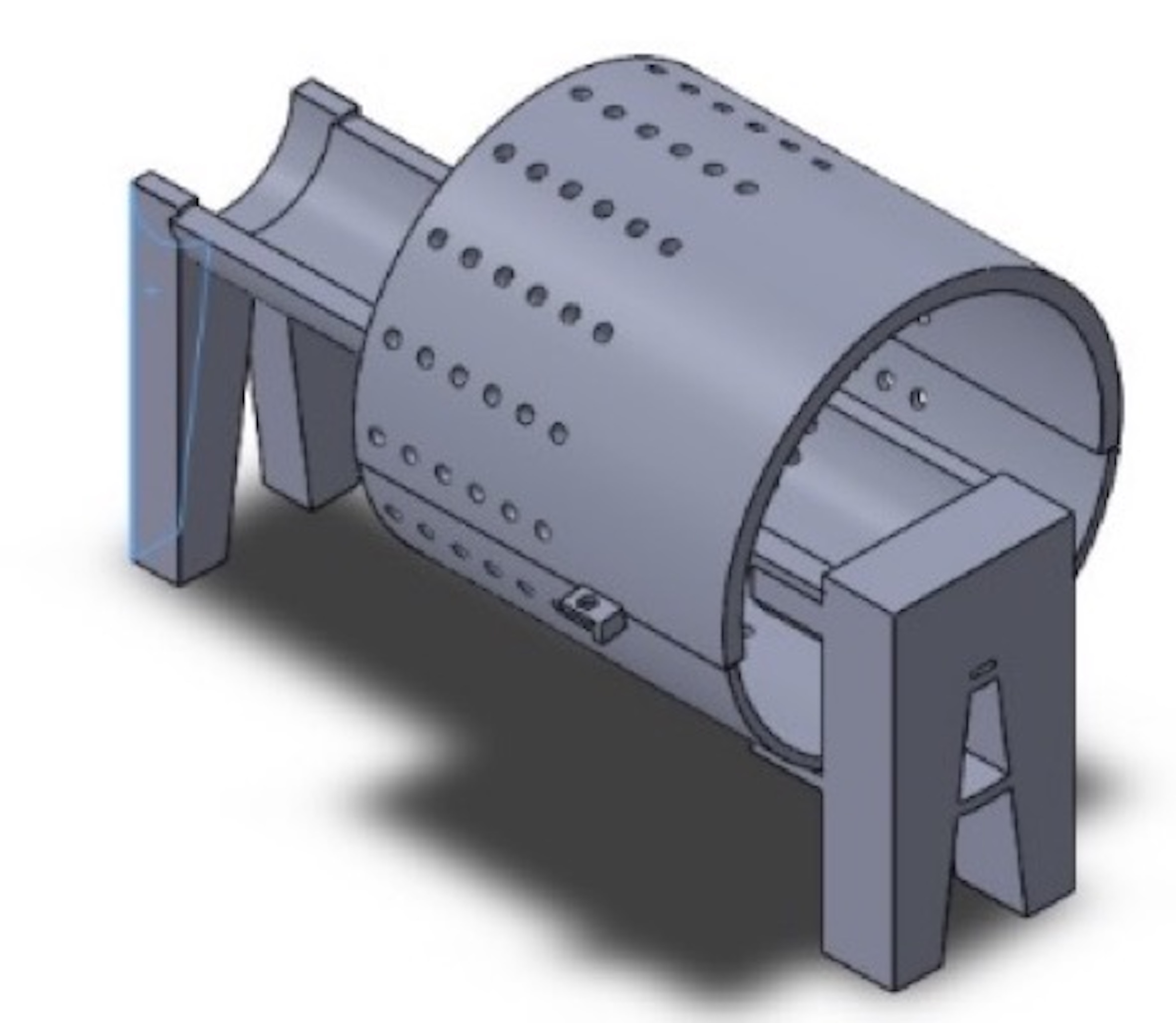

Supplement: Supplementary file 2 [file Image2.JPEG]
